# Improved One- and Multiple-Photon Excited Photoluminescence from Cd^2+^-Doped CsPbBr_3_ Perovskite NCs

**DOI:** 10.3390/nano12010151

**Published:** 2022-01-01

**Authors:** Ivan D. Skurlov, Wenxu Yin, Azat O. Ismagilov, Anton N. Tcypkin, Haohang Hua, Haibo Wang, Xiaoyu Zhang, Aleksandr P. Litvin, Weitao Zheng

**Affiliations:** 1 Laboratory of Optics of Quantum Nanostructures, ITMO University, 197101 St. Petersburg, Russia; sky_id@itmo.ru; 2 Key Laboratory of Automobile Materials, College of Materials Science and Engineering, Jilin University, Changchun 130012, China; wenxuyin0108@163.com (W.Y.); huahaohang0520@163.com (H.H.); wanghaibo@jlu.edu.cn (H.W.); wtzheng@jlu.edu.cn (W.Z.); 3 Laboratory of Quantum Processes and Measurements, ITMO University, 197101 St. Petersburg, Russia; ismagilov.azat@itmo.ru (A.O.I.); tsypkinan@itmo.ru (A.N.T.)

**Keywords:** perovskite, nanocrystal, doping, nonlinear optical properties, photoluminescence, two-photon absorption

## Abstract

Metal halide perovskite nanocrystals (NCs) attract much attention for light-emitting applications due to their exceptional optical properties. More recently, perovskite NCs have begun to be considered a promising material for nonlinear optical applications. Numerous strategies have recently been developed to improve the properties of metal halide perovskite NCs. Among them, B-site doping is one of the most promising ways to enhance their brightness and stability. However, there is a lack of study of the influence of B-site doping on the nonlinear optical properties of inorganic perovskite NCs. Here, we demonstrate that Cd^2+^ doping simultaneously improves both the linear (higher photoluminescence quantum yield, larger exciton binding energy, reduced trap states density, and faster radiative recombination) and nonlinear (higher two- and three-photon absorption cross-sections) optical properties of CsPbBr_3_ NCs. Cd^2+^ doping results in a two-photon absorption cross-section, reaching 2.6 × 10^6^ Goeppert-Mayer (GM), which is among the highest reported for CsPbBr_3_ NCs.

## 1. Introduction

Doping of cesium lead halide perovskites has become a novel approach to tuning their optoelectronic properties and enhancing their stability [1,2,3,4]. Doping may enhance their luminosity and promote charge carrier transport and crystal phase stabilization [2]. Different divalent metal ions including Mn^2+^, Sn^2+^, Ca^2+^, Zn^2+^, Cu^2+^, and Ni^2+^ have been used for isovalent B-site doping (where ABX_3_ is a general structural formula) of cesium lead halide perovskite nanocrystals (NCs) [5,6,7,8,9,10,11,12,13,14,15,16,17]. B-site doping is also considered a new approach to achieving better characteristics of devices based on perovskite NCs, and that was demonstrated for CsPbBr_3_ NCs, which are important nanomaterials to produce green-emitting light-emitting diodes (LEDs) [12,13,14,15,16,17]. Recently, the doping of CsPbBr_3_ NCs with Mn^2+^ [18], Sn^2+^ [19], Ce^3+^ [20], Rb^+^ [21], and Co^2+^ [22] has been used to enhance the external quantum efficiency (EQE) and maximum luminance of the LEDs. Moreover, the doping may facilitate better environmental stability of the device [18] and optimization of the energy band diagram [23].

Metal halide perovskites can now be considered a new platform for nonlinear optics [24]. For instance, they demonstrate enormous values of nonlinear absorption cross-sections. The study of the processes involving simultaneous absorption of multiple photons is of great importance for numerous applications, including high-resolution microscopy [25,26] and biomedical imaging [27,28]. Despite the great practical interest in these processes, their utilization is limited due to the lack of highly efficient nonlinear materials, and the employment of perovskites in this field is of tremendous importance. B-cite doping is considered to be an effective tool for the further optimization of nonlinear optical responses from metal halide perovskite NCs [29,30]; however, very few reports have been published on this topic. Ketavath et al., recently showed that 0.08–0.1% Ni^2+^ doping of 2D CsPbBr_3_ NCs induced a ~2.5-fold increase in the two-photon absorption (2PA) cross-section [31]. He et al., studied the influence of Mn^2+^ doping on the nonlinear optical properties of CsPbCl_3_ NCs [32] and nanoplatelets (NPLs) [33]. They reported the wavelength-dependent 2PA cross-section of Mn^2+^-doped CsPbCl_3_ NCs with a maximum value up to ~3.2 × 10^5^ GM (1 GM = 10^−50^ cm^−4^ s photon^−1^) [32].

Cd^2+^ doping is an important example of engineering of perovskite NC properties. Cd^2+^ doping of CsPbBr_3_ NCs was first reported by van der Stam and co-workers, who proposed a post-synthetic cation exchange reaction to replace some of the Pb^2+^ cations [11]. They showed that the doping caused a blue shift of optical transitions, induced by lattice contraction, while preserving a high photoluminescence quantum yield (PLQY) and narrow full width at half maximum (FWHM) of the PL band. A similar observation was recently made by Zhao et al., who synthesized Cd^2+^ doped CsPbBr_3_ NCs inside a borosilicate glass matrix [34]. As an alternative approach, Mondal and co-workers demonstrated the post-synthetic treatment of CsPbCl_3_ NCs with CdCl_2_ that resulted in the tremendous growth of PLQY [35]. Very recently, Xie et al., demonstrated that post-synthetic surface treatment of CsPbBr_3_ NCs with CdX_2_ precursors enlarged their PLQY from 85% to 92% [36].

Although these results point to the undeniable effect of Cd^2+^ doping on all-inorganic NC optical properties, there is no information regarding its influence on their nonlinear optical properties. Here, we developed methods for obtaining Cd^2+^-doped perovskite NCs by direct synthesis using a mixture of PbBr_2_ and CdBr_2_ precursors. The investigation of the temperature dependencies of the PL responses allowed us to reveal additional features induced by Cd^2+^ doping. For the first time, we demonstrate that Cd^2+^ doping modifies nonlinear optical responses of CsPbBr_3_ NCs: Cd^2+^ doped CsPbBr_3_ NCs demonstrated bright one- and multi-photon excited emission, while a two-photon absorption cross-section reached 2.6 × 10^6^ GM. First, we describe the influence of doping on the structural and linear optical properties of CsPbBr_3_ NCs that allow the fabrication of an LED with a maximum luminance of 24,221 cd·m^−2^, and then we demonstrate the improvement of nonlinear optical responses studied by means of two- and three-photon excited photoluminescent spectroscopy.

## 2. Materials and Methods

Materials. Lead bromide (PbBr_2_, 99%), cesium carbonate (Cs_2_CO_3_, 99.95%), octadecene (ODE, 90%), oleic acid (OA, 90%), cadmium bromide tetrahydrate (CdBr_2_∙4H_2_O, 98%), and oleylamine (OlAm, 80–90%) were purchased from Aladdin (Shanghai, China). Hexane, ethanol, and acetone were purchased from Sinopharm Chemical Reagent Co., Ltd. (Shanghai, China). Poly(3,4-ethylenedioxythiophene): polystyrene sulphonate (PEDOT:PSS), Poly[bis(4-phenyl) (4-butylphenyl) amine] (Poly-TPD) and 2,2′,2″-(1,3,5-Benzinetriyl)-tris(1-phenyl-1-Hbenzimidazole) (TPBi) were purchased from Xi’an Polymer (Xi’an, Shannxi, China). Lithium fluoride (LiF) was purchased from Lumtec (New Taipei City, Taiwan).

Preparation of cesium oleate (Cs-OA) solution. In brief, Cs_2_CO_3_ (0.814 g), OA (2.5 mL), and ODE (30 mL) were loaded into a 100 mL three-neck flask. The flask was degassed at 120 °C for 60 min and then switched to N_2_ flow. Finally, the flask was heated up to 150 °C until the Cs_2_CO_3_ completely dissolved.

Preparation of CsPbBr_3_ and CsPb_0.78_Cd_0.22_Br_3_ NCs. Briefly, PbBr_2_ (0.376 mmol) and ODE (10 mL) were added to a 50 mL three-neck round bottom flask. The solution was degassed at 120 °C for 60 min under vacuum and then switched to N_2_ flow. After injection of OA (1 mL) and OlAm (1 mL) the temperature was increased to 180 °C. Subsequently, as-synthesized Cs-OA (1 mL) was promptly injected to the flask. After 5 s, an ice bath was used to stop the reaction. CsPb_0.78_Cd_0.22_Br_3_ NCs were synthesized by adding 0.188 mmol of CdBr_2_·4H_2_O at the beginning of reaction while keeping the rest of the protocol intact.

Purification. First, the crude solution was purified by centrifugation at 5000 RPM for 10 min; the supernatant was discarded, and the precipitate was re-dispersed in hexane to form stable colloidal solutions. Second, ethyl acetate was introduced into the colloidal solutions at a volume ratio of 1:1, and then the mixture was centrifuged for 10 min at 10,000 RPM. Lastly, the final precipitate was dispersed in 2 mL hexane for LED fabrication.

LED fabrication. Glass substrates coated with indium tin oxide (ITO) were first sonicated consecutively with ethanol and acetone for 20 min each. To increase the work function, ITO substrates were treated with UV-ozone for 15 min. Then, PEDOT: PSS (25 nm thickness) was spin-coated on the ITO substrates at a speed of 4000 RPM for 40 s and then annealed at 150 °C for 15 min in ambient air. Subsequently, these substrates were transferred into a glovebox. The Poly-TPD solution (dissolved in chlorobenzene, 8 mg/mL, 30 nm) was spin coated onto the PEDOT: PSS film at a speed of 4000 RPM for 40 s and annealed at 150 °C for 15 min. The washed CsPb_0.78_Cd_0.22_Br_3_ NCs (~35 nm thickness) were spin coated onto the Poly-TPD film at 1500 RPM for 40 s. Finally, TPBi (18 nm), LiF, and Al (LiF/Al, 55 nm) electrodes were deposited by thermal evaporation in a vacuum deposition chamber of ~3 × 10^−6^ Torr.

Characterization. UV-VIS absorption was measured using a Shimadzu UV-1800 (Shimadzu corporation, Kyoto, Japan) spectrophotometer. A JEM-2100F (JEOL Ltd., Tokyo, Japan) transmission electron microscope (TEM) was used to analyze the morphology of the NCs. X-ray diffraction (XRD) patterns were acquired using a Bruker D8 Advance X diffractometer (Cu Kα, *λ* = 1.5406 Å). X-ray Photoelectron Spectroscopy (XPS) was performed on an ESCALAB 250 (Thermo Fisher Scientific Inc., Waltham, MA, USA) spectrometer with a mono X-Ray source Al Kα excitation (1486.6 eV). Ultraviolet photoelectron spectra (UPS) were collected using a PREVAC (PREVAC, Rogów, Poland) system. A Keithley 2400 (Keysight technologies, Santa Rosa, CA, USA) source meter and a PR-750 Spectroradiometer (Photo Research Inc., Chatsworth, CA, USA) were used to acquire the current density–luminance–voltage (J-V-L) characteristics. The molar concentration of the nanocrystals in the solution was obtained from the absorption spectra; see Appendix A for details.

PL spectroscopy. PL spectra were measured using a custom-built setup based on an Acton-2500 monochromator equipped with an Andor iDus 401A CCD camera (Andor Technology Ltd., Belfast, Northern Ireland) [37,38,39]. To avoid any thermal effects, the samples were excited with unfocused 80 µW 405 nm laser (Lasever Inc., Ningbo, China) excitation passed through a 0.8 mm diaphragm. The obtained spectra were corrected using the blackbody radiation source (Thorlabs SLS201/M, Thorlabs, Newton, NJ, USA). For the measurements at decreased temperatures, NCs were added to 8 wt% PMMA solution in toluene under an inert atmosphere and were left stirring for 1 h at 1000 RPM. The solution was dropped onto a precleaned glass substrate and spin-coated at 2000 RPM. The optical density of the films did not exceed 0.1 at 400 nm. The samples were transferred to a Linkam THMS600 Microscope Stage cryostat (Linkam scientific instruments, Tadworth, UK). For the transient measurements, a MicroTime 100 microscope (PicoQuant, Berlin, Germany) employing time-correlated single-photon counting was used. The microscope was equipped with 3× objective lens and a 50 ps pulse diode laser head operated at 405 nm. PL lifetimes were found to be dependent on the excitation power density, as was previously reported [40]. The excitation power density was maintained below the value at which the dependence of PL lifetimes on excitation intensity occurred. The IRF was measured and deconvoluted from the decay curves. After the deconvolution, the PL decays were fitted by the 3-exponential decay law.

2-photon excited PL measurements. Nanocrystals were dispersed in toluene in 1-mm-thick optical glass cuvettes. Two-photon PL was excited using an Avesta femtosecond laser system (Avesta project Ltd., Troitsk, Moscow, Russia) with a peak center at 800 nm, pulse width of ~30 fs, 1 kHz repetition rate, and power density ranging from 0.035 W/cm^2^ to 1.150 W/cm^2^. PL was detected using a USB4000-UV-VIS-ES (Ocean Insight, Largo, FL, USA) spectrometer [41]. The obtained spectra were corrected using the blackbody radiation source (Thorlabs SLS201/M, Thorlabs, Newton, NJ, USA). Rhodamine 6G in ethanol was used as a standard for the 2PA cross-section calculation. Rhodamine 6G cross-section was taken to be 128 ± 84 GM (10^−50^ cm^−4^·s·photon^−1^) [42,43,44]. A detailed description of the two-photon excited PL analysis can be found in Appendix B.

3-photon excited PL measurements. An optical parametric amplifier (OPA-TOPAS, Light Conversion, Vilnius, Lithuania) was used as an excitation source for the generation of 3-photon excited PL from perovskite NC solutions in toluene. The femtosecond pump radiation from a regenerative Ti-sapphire amplifier (pulse duration 35 fs, energy 2.2 mJ, 1 kHz repetition rate, 800 nm central wavelength) was used as a pump radiation for OPA. Optical radiation from OPA in the range 1300–1560 nm and under a pulse duration of 60 fs passed through quartz cuvette with sample solutions. The diameter of the laser beam was 5 mm, and the excitation power density varied from 0.35 W/cm^2^ to 1.52 W/cm^2^. To measure the dependence of the integrated PL intensity vs. power density, the excitation wavelength was fixed at 1300 nm, and an optical attenuator with neutral filters was used to change the excitation power. For the wavelength-dependent measurements, the pulse energy was fixed at 250 ± 20 μJ. The PL signal was detected using a USB4000-UV-VIS-ES (Ocean Insight, Largo, FL, USA) spectrometer. 3PA absorption cross-section was calculated analogous to the two-photon excitation (see Appendix B for the details) with Rhodamine 6G in ethanol as a reference (3PA cross section of 3 × 10^−80^ cm^6^ s^2^ [45].

## 3. Results

### 3.1. Synthesis and Characterization of NCs

**Figure 1:**
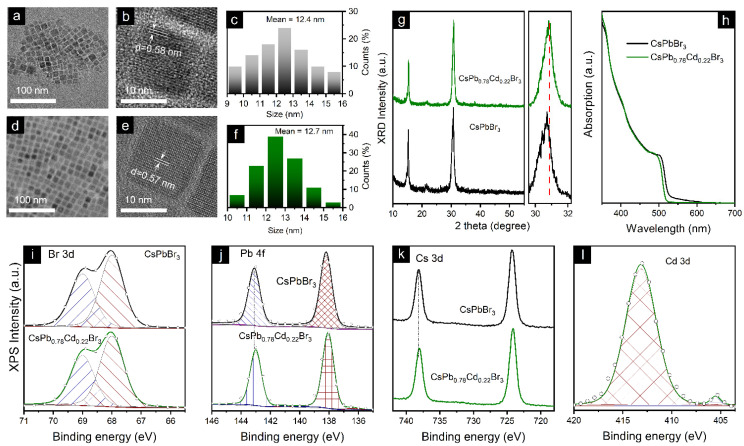
TEM images of (**a**) CsPbBr_3_ and (**d**) CsPb_0.78_Cd_0.22_Br_3_ NCs; their corresponding HR-TEM images are exhibited in (**b**,**e**). (**c**,**f**) are particle size distribution histograms of CsPbBr_3_ and CsPb_0.78_Cd_0.22_Br_3_ NCs; their corresponding XRD patterns and UV−vis absorption spectra are shown in (**g**,**h**), respectively. (**i**–**l**) XPS spectra for the (**a**) Br, (**b**) Pb, (**c**) Cs, and (**d**) Cd elements collected for the CsPbBr_3_ and CsPb_0.78_Cd_0.22_Br_3_ NCs.

The morphology and crystal structure of the as-synthesized CsPbBr_3_ NCs and Cd^2+^-doped CsPbBr_3_ (CsPb_0.78_Cd_0.22_Br_3_, the element content was confirmed by X-ray photoelectron spectroscopy) NCs were investigated by transmission electron microscopy (TEM). As shown in Figure 1a,d, the TEM image of CsPb_0.78_Cd_0.22_Br_3_ NCs exhibited a more uniform morphology and narrower size distribution than CsPbBr_3_ NCs, as clearly seen from the particle size distribution histograms (Figure 1c,f). Both samples were highly crystalline, which can be confirmed by the ordered crystal lattices in high-resolution TEM (HRTEM) images of Figure 1e. The obvious lattice fringes were identified to be 5.8 and 5.7 Å, which is in good agreement with the (100) crystal facets of the CsPbBr_3_ cubic phase. As for the reduction in lattice spacing in CsPb_0.78_Cd_0.22_Br_3_ NCs, it can be attributed to the lattice contraction derived from the Cd^2+^ doping. To further characterize the crystal structure, we measured X-ray diffraction patterns (XRD); the results are presented in Figure 1g. The two main peaks located at ~14.8° and 30.2° were assigned to the (100) and (200) planes of CsPbBr_3_ cubic phase, respectively. In contrast to CsPbBr_3_ NCs, the characteristic peaks of CsPb_0.78_Cd_0.22_Br_3_ NCs showed an obvious shift to higher angles without introducing any extra peaks, which indicates that the Cd^2+^ incorporation did not change the crystal structure but only contracted the lattice spacing, corresponding to the HRTEM results. To verify the effect on optical properties, the absorption spectra of these samples were measured and are summarized in Figure 1h. The characteristic absorption peak shifted from 501 nm to 495 nm after Cd^2+^ doping, and the absorption largely decreased in the 520–600 nm range, indicating the reduction of the trap state density in CsPb_0.78_Cd_0.22_Br_3_ NCs.

To identify the influence of Cd^2+^ doping on the chemical states of constituting elements in the perovskite NC film, we performed X-ray photoelectron spectroscopy (XPS) measurements of CsPbBr_3_ and CsPb_0.78_Cd_0.22_Br_3_ NC films. Figure 1i–l shows XPS spectra for the Br, Pb, Cs, and Cd elements, respectively, all calibrated with C 1 s. No peak shift was observed for Br 3d, while the Pb 4f and Cs 3d peaks shifted to higher binding energy in the CsPb_0.78_Cd_0.22_Br_3_ NC film compared to the CsPbBr_3_ NC film. The result is consistent with the crystal lattice contraction observed in the HRTEM images and XRD patterns. As expected, CsPb_0.78_Cd_0.22_Br_3_ NC films demonstrated a noticeable Cd 3d peak, and the elemental ratio of Cd:Pb was around 0.29:1.

### 3.2. One-Photon Excited PL

**Figure 2:**
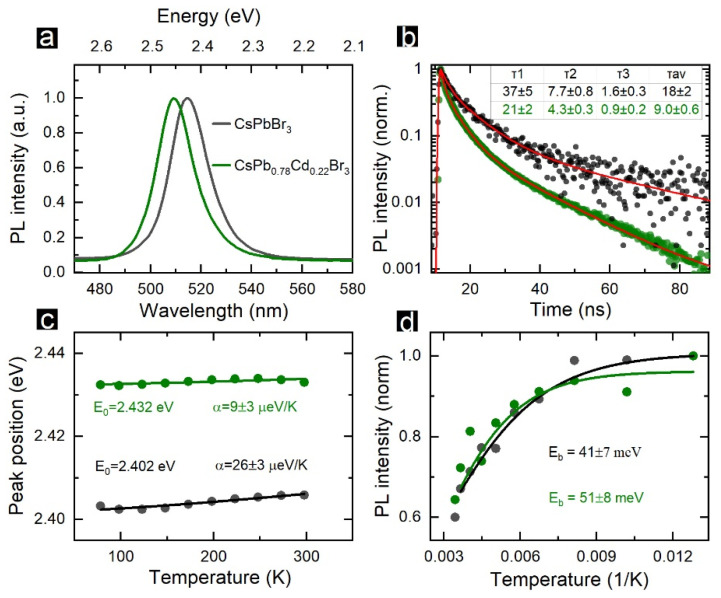
One-photon excited PL from CsPbBr_3_ (black) and CsPb_0.78_Cd_0.22_Br_3_ (green) NCs. (**a**) PL spectra recorded under 405 nm laser excitation. (**b**) PL decay curves recorded under pulsed 405 nm laser excitation; red lines show 3-exponential fitting, and the estimated decay times constants are listed in the table along with the average PL decay times. (**c**) Temperature dependencies of the PL peak position; solid lines show Varshni fitting. (**d**) Temperature dependencies of integrated PL intensity; solid lines show Arrhenius-type fitting.

The PL spectra for CsPbBr_3_ (black) and CsPb_0.78_Cd_0.22_Br_3_ (green) NCs in toluene are shown in Figure 2a. The Cd^2+^ doping induced a 25-meV blue shift of the PL peak position. The blue shift of PL peak position and absorption onset (Figure 1h) are typical for the doping of perovskite NCs by guest cations with smaller ionic radii [3]. The measured change of the bandgap fitted well with the theoretical predictions reported by Navas et al., for Cd^2+-^doped MAPbI_3_ NCs [46]. Van der Stam et al. [11] recently demonstrated that introducing smaller cations, including Cd^2+^, induced a parent NC lattice contraction. A small shift of the band-edge emission was recently observed for Cd^2+^-doped CsPbCl_3_ NCs [47], which indicates that Cd^2+^ ions diffuse into the NC volume rather than passivate their surface. Both spectra shown in Figure 2a had similar FWHMs, i.e., 90 and 91 meV. After the Cd^2+^ doping, the PLQY increased from 75% to 90%. The PL decay for pristine and doped NCs is shown in Figure 2b. The decay curves were fitted by a 3-exponential function. The averaged PL lifetime decreased from 18 ± 2 to 9.0 ± 0.5 ns after the doping, which corresponds well with the data obtained by Van der Stam et al., for Cd^2+^-doped CsPbBr_3_ NCs [11]. Since we observed both a decrease in the PL decay time and an increase in the PLQY, this points to an increase in the radiative recombination rate and not the appearance of additional fast nonradiative recombination channels.

The analysis of the temperature dependencies of PL spectra revealed more differences between the pristine and doped NCs. Figure 2 shows the temperature dependencies of the PL peak positions and their approximation with a Varshnii function (Equation (1)) [48].

(1) $$E_{G}\left(T\right)=E_{G0}-\alpha \frac{T^{2}}{T+\beta },$$

where *E_G_*_0_ is the bandgap (PL peak position) at 0 K, α is the coefficient of the temperature shift, and *β* is the Debye temperature, i.e., 224.8 K [49]. The peak position temperature shift coefficient *α* was 26 ± 3 µeV/K for CsPbCl_3_ NCs, which is almost an order smaller than that reported in Refs. [40,50,51]. Since many physical mechanisms contribute to a temperature shift of the bandgap for nanosized semiconductors, many factors may influence the determined *α* value, and the reports for different synthetic procedures may vary in a wide range [52]. The CsPb_0.78_Cd_0.22_Br_3_ NCs demonstrated an even smaller *α* value, 9 ± 3 µeV/K. Such a negligible temperature shift of the PL band is beneficial for the development of LEDs with stable color purity [53]. Both types of NCs demonstrated an increase in PL intensity with a temperature decrease (Figure 2d). Fitting the integrated PL intensity vs. temperature with Arrhenius-type equation (Equation (2)) allowed us to determine the exciton binding energy (*E_b_*) in perovskite NCs [54].

(2) $$I_{PL}=\frac{I_{0}}{\left(1+A\times e^{-\frac{E_{b}}{k_{B}\cdot T}}\right)}$$

where *I*_0_ is the integrated PL intensity at 0 K, *k_B_* is the Boltzmann constant, and *E_b_* is the binding energy. *E_b_* was 41 ± 7 meV for the pristine CsPbBr_3_ NCs. This value is very close to those recently determined by Yuan et al., using the same experimental approach [51] and estimated by Protesescu et al., within the effective mass approximation [55]. The CsPb_0.78_Cd_0.22_Br_3_ NCs had a higher *E_b_* value, 51 ± 8 meV, which makes them attractive candidates to be used in perovskite-based LEDs [56,57].

### 3.3. LEDs

**Figure 3:**
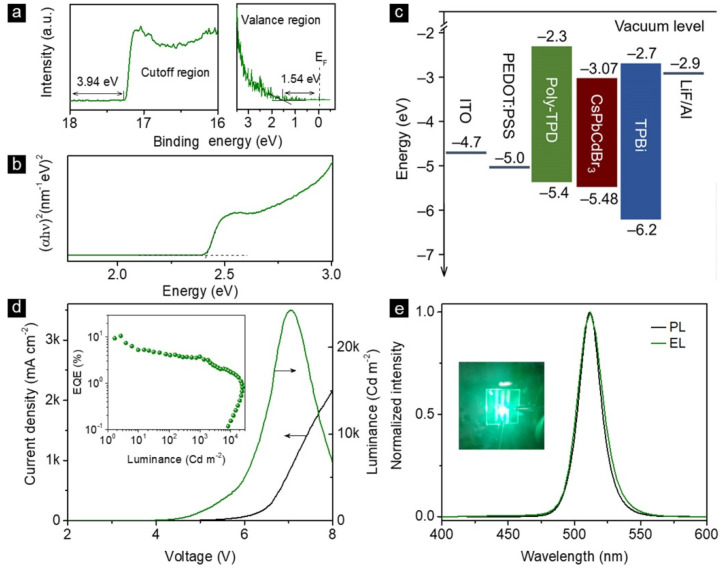
(**a**) UPS spectra for the cutoff (left) and valence (right) regions and (**b**) Tauc plot of the CsPb_0.78_Cd_0.22_Br_3_ NCs film; (**c**) overall energy band diagram of the CsPb_0.78_Cd_0.22_Br_3_ NC-based LED, (**d**) current density and brightness vs. driving voltage of the CsPb_0.78_Cd_0.22_Br_3_ NC-based LED, (**e**) normalized PL and EL spectra for CsPb_0.78_Cd_0.22_Br_3_ NCs in the film and LEDs; the inset is a photograph of the working LED.

To demonstrate their practical applicability, the CsPb_0.78_Cd_0.22_Br_3_ NCs were adopted as emitters to fabricate LEDs. The lowest unoccupied molecular orbital (LUMO) and the highest occupied molecular orbital (HOMO) were determined by the ultraviolet photoelectron spectroscopy (UPS, Figure 3a) of CsPb_0.78_Cd_0.22_Br_3_ NC film [58]. The Tauc plot of the CsPb_0.78_Cd_0.22_Br_3_ NC film (Figure 3b) revealed a bandgap of 2.41 eV [59]. Thus, the LUMO and HOMO values of CsPb_0.78_Cd_0.22_Br_3_ NCs were confirmed to be −3.07 and −5.48 eV, respectively. The conventional multilayered LED was fabricated using patterned indium−tin oxide (ITO) as the anode, poly(ethylenedioxythiophene):polystyrenesulfonate (PEDOT:PSS, 25 nm) film and poly(*N*,*N*′-bis(4-butylphenyl)-*N*,*N*′-bis(phenyl)-benzidine) (poly-TPD, 40 nm) film as the hole transporting layer (HTL), CsPb_0.78_Cd_0.22_Br_3_ NC film (40 nm) as the emitting layer, 1,3,5-tris(*N*-phenylbenzimidazol-2-yl) benzene (TPBi, 40 nm) film as the electron-transporting layer (ETL), and LiF/Al as the cathode. The energy band diagram for all functional layers of LED is given in Figure 3c. Figure 3d demonstrates the current density–voltage (J–V) and luminance–voltage (L–V) curves of the LEDs, which showed a peak luminance of 24,221 Cd·m^−2^ under the working bias of 7.0 V. The peak EQE of 10.6% was achieved at 0.3 mA cm^−2^ (inset of Figure 3d). Normalized PL and electroluminescence (EL) spectra of a typical LED are given in Figure 3e. Apparently, the EL originated from CsPb_0.78_Cd_0.22_Br_3_ NCs without noticeable contribution from any charge transport layers, indicating that the perovskite NCs served as the exciton recombination centers for the device, and a balanced charge transport was achieved. The device PL and EL both exhibited emission peaks at 511 nm. The inset of Figure 3e is the image of a working LED.

### 3.4. Multiphoton-Induced PL

**Figure 4:**
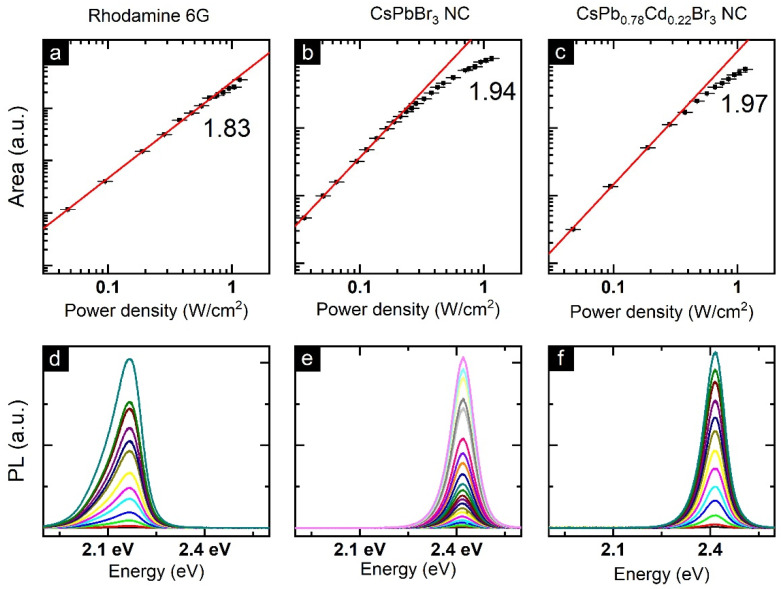
The log–log plots of integrated PL area vs. power density for Rhodamine 6G (**a**), CsPbBr_3_ NCs (**b**), and CsPb_0.78_Cd_0.22_Br_3_ NCs (**c**). (**d**–**f**) corresponding 2PA-excited PL spectra measured at different excitation power densities.

Two-photon excited PL spectra of perovskite NCs were recorded using non-resonant 800 nm laser excitation. To estimate the type of multi-photon process, we measured NC PL spectra with different excitation power densities. Figure 4a–c demonstrates the log–log plots of integrated PL intensity vs. excitation power density for the Rhodamine 6G (used as a reference), CsPbBr_3_ NCs, and CsPb_0.78_Cd_0.22_Br_3_ NCs, respectively. Nearly quadratic dependencies of integrated PL intensity vs. power density clearly indicate the two-photon absorption (2PA) process [60]. At higher excitation power density, the integrated 2PA-excited PL intensity demonstrated a saturation-like behavior as previously reported for the MAPbBr_3_ films [61] and 2D (PEA)_2_PbI_4_ perovskites [62]. We can attribute this observation to the influence of strong laser radiation exposure, which can lead to perovskite NС partial destruction caused by heating. It should also be noted that CsPb_0.78_Cd_0.22_Br_3_ NCs showed a smaller deviation from the theoretical power law, which may indicate their improved stability under laser radiation.

The 2PA absorption cross-sections were calculated using Rhodamine 6G as a standard (see experimental section and Appendix B). The reported NC values of third-order nonlinear optical parameters strongly depend on their stoichiometry [63], size [64], and shape [65]. The value obtained for the pristine CsPbBr_3_ NCs (3.2 ± 1.9) × 10^5^ GM was very close to those recently reported (see Table 1) [64,65,66,67,68]. CsPb_0.78_Cd_0.22_Br_3_ NCs demonstrated an almost one-order enhanced value of the 2PA cross-section, and the obtained value is among the highest reported for CsPbBr_3_ NCs [69,70].

**Figure 5:**
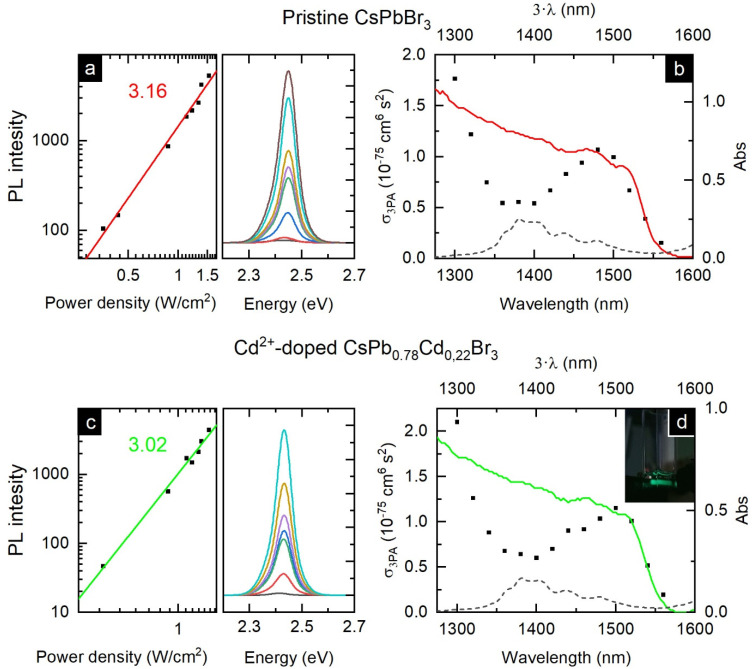
Three-photon excited photoluminescence of the studied NC. The log–log plots of the integrated PL area vs. power density for CsPbBr_3_ NCs (**a**) and CsPb_0.78_Cd_0.22_Br_3_ NCs (**c**). On the side are corresponding 3PA-excited PL spectra measured at different excitation power densities. 3PA absorption cross-section spectral dependencies for (**b**) CsPbBr_3_ NCs and (**d**) CsPb_0.78_Cd_0.22_Br_3_ NCs (on the inset is the photo of the PL from the CsPb_0.78_Cd_0.22_Br_3_ NC solution when excited at 1560 nm). Dashed lines in (**b**,**d**) are the absorption of toluene at 1300–1600 nm, while colored lines show corresponding linear absorption drawn as a function of Abs(3·λ).

To study the 5th-order nonlinear optical response, three-photon absorption (3PA)-induced PL was investigated using the wavelength-tunable excitation in the 1300–1560 nm spectral range. First, the excitation wavelength was fixed at 1300 nm, and the integrated PL intensity vs. excitation power dependencies for the pristine and doped perovskite NCs were measured (Figure 5a,c). For both samples, the dependencies were similar to a cubic function, indicating the 3-photon excited process solely [74]. To calculate the 3PA cross-sections, Rhodamine 6G was used as a standard. Similar to the results obtained for the 2PA excited PL, the 3PA cross-section was higher for CsPb_0.78_Cd_0.22_Br_3_ NCs (2.1 ± 0.7 vs. 1.7 ± 1.0 ×10^−75^ cm^6^ s^2^ photon^−2^). Spectral tuning of the used excitation allowed us to obtain the wavelength-dependencies of the 3PA cross-section for the pristine CsPbBr_3_ and doped CsPb_0.78_Cd_0.22_Br_3_ NC (Figure 5b,d). For both samples, the wavelength dependencies of the 3PA cross-sections well matched the linear absorption spectra of the NC solutions, except the region of intense absorption of toluene (1340–1440 nm). This observation is in line with previously reported wavelength dependencies obtained for both 2PA and 3PA in CsPbBr_3_ NCs [64,68].

## 4. Conclusions

To conclude, we demonstrate that the engineering of the B-site in metal halide perovskite NCs has great potential for the improvement of their optical properties. Importantly, we revealed that this strategy may induce a huge change in their nonlinear optical responses. The insertion of Cd^2+^ into CsPbBr_3_ NCs resulted in an improvement of their morphological and optical properties, including greater exciton binding energy of 51 meV, reduced trap state density, faster radiative recombination, and high photoluminescence quantum yield reaching 90%. An even more significant improvement was associated with a change in the nonlinear optical response of the Cd^2+^-doped CsPbBr_3_ NCs. The TPA cross-section of 2.6 × 10^6^ GM was evaluated, which is among the highest reported for CsPbBr_3_ perovskite NCs. The findings demonstrate that the perovskite NCs with a rationally engineered B-site have great potential for light-emission and nonlinear optical applications.

## Figures and Tables

**Table 1:** Pristine and volume-normalized 2PA and 3PA cross-sections for the CsPbBr_3_ NCs, nanoplatelets (NP), nanorods (NR), and nanowires (NW).

Sample	σ_2PA_ (GM)	Volume Normalized σ_2PA_ GM/nm^3^)	σ_3PA_ (10^−80^ cm^6^ s^2^ Photon^−2^)	Volume Normalized σ_3PA_ (10^−80^ cm^6^ s^2^ Photon^−2^ nm^−3^)	Ref.
CsPbBr_3_ NC 12.4 nm	(3.2 ± 1.9) ×10^5^ (800 nm, 30 fs)	171 ± 112	(1.7 ± 1.0) ×10^5^ (1300 nm, 30 fs)	89 ± 18	This work
CsPb_0.78_Cd_0.22_Br_3_ NC 12.7 nm	(2.6 ± 0.8) × 10^6^ (800 nm, 30 fs)	1265 ± 830	(2.1 ± 0.7) ×10^5^ (1300 nm, 30 fs)	102 ± 21	This work
CsPbBr_3_ NC 7.3 nm	1.2 × 10^5^ (720 nm 100 fs)	308	2.8 × 10^4^ (1200 nm 100 fs)	72	[68]
CsPbBr_3_ NP	2.28 × 10^5^ (720 nm 100 fs)	1562	1.05 × 10^5^ (1200 nm 100 fs)	720	[68]
CsPbBr_3_ NC 9 nm	2 × 10^6^ (800 nm 100 fs)	2743	1.0 × 10^6^ (1200 nm 100 fs)	1372	[71]
CsPbBr_3_ NC 7 nm	1.8 × 10^5^ (700–1000 nm 100 fs)	525	9.1 × 10^5^ (1000–1500 nm 100 fs)	2653	[63]
CsPbBr_3_ NR	1.5 × 10^6^ (800 nm 100 fs)	221	2.7 × 10^5^ (1300 nm 100 fs)	40	[72]
CsPbBr_3_ NC 12 nm	9.8 × 10^5^ (800 nm 50 fs)	567			[65]
CsPbBr_3_ NC 9 nm	1.2 × 10^5^ (800 nm 100 fs)	164			[66]
CsPbBr_3_ NC 12.4 nm	2.2 × 10^5^ (800 nm)	115			[67]
CsPbBr_3_ NC 9 nm	2.7 × 10^6^ (800 nm 90 fs)	3704			[69]
CsPbBr_3_ NC 4.6 nm	1.6 × 10^4^ (800 nm 120 fs)	164			[64]
CsPbBr_3_ NC 5.2 nm	2.9 × 10^4^ (800 nm 120 fs)	206			[64]
CsPbBr_3_ NC 6.9 nm	6.1 × 10^4^ (800 nm 120 fs)	186			[64]
CsPbBr_3_ NC 9.4 nm	1.8 × 10^5^ (800 nm 120 fs)	217			[64]
CsPbBr_3_ NC 11.4 nm	4.55 × 10^5^ (800 nm 120 fs)	307			[64]
CsPbBr_3_ NC 28 nm	8.1 × 10^4^ (800 nm 100 fs)	4			[73]
CsPbBr_3_ NP	4.2 × 10^5^ (800 nm 100 fs)	300			[73]
CsPbBr_3_ NW	2.3 × 10^5^ (800 nm 100 fs)	12			[73]

## Data Availability

All required data is provided within the manuscript.

## Appendix A. Calculation of Perovskite NC Molar Concentration

The perovskite NC extinction coefficient (*ε*) at 400 nm was calculated from the following equation:

(A1) $$\varepsilon =\frac{D}{C_{M}l}=\frac{m_{NC}}{C_{mass}}\frac{D}{l}=\frac{N_{A}\times V_{NC}\times \rho }{C_{mass}}\frac{D}{l}$$

where *D* is the absorbance of the sample at 400 nm; *C_M_*—NC molar concentration; *l*—cuvette length; *m_NC_*—molar mass of the single NC; *C_mass_*—mass concentration; *N_A_*—the Avogadro number; *V_NC_*—NC volume (determined from the known NC size); *ρ*—the NC density. NC density was calculated by normalizing the crystalline cell mass to the cell volume:

(A2) $$\rho =\frac{m_{cell}}{V_{cell}}=\frac{m_{cell}}{r^{3}}$$

where *m_cell_* and *V_cell_* are the respective cell mass and cell volume, *r* is the edge length, determined from TEM (see Figure 1). Cell mass was calculated from known NC stoichiometry. The resulting densities were 4.935 g/cm^3^ for CsPbBr_3_ and 5.012 g/cm^3^ for CsPb_0.78_Cd_0.22_Br_3_. Increased NC density in doped NCs arises from the lattice contraction observed from TEM (see Figure 1b,e). Mass concentration of the solution was determined from the weight of thoroughly washed and dried NCs.

The ε values for the CsPbBr_3_ and CsPb_0.78_Cd_0.22_Br_3_ NCs were (3.63 ± 0.18) × 10^7^ mol^−1^cm^−1^ and (3.57 ± 0.09) × 10^7^ mol^−1^cm^−1^, respectively. To verify the calculated extinction coefficients, we compared the values with the ones that were obtained for the pristine CsPbBr_3_ using Equation (A3) [75].

(A3) $$\varepsilon =2.42\times 10^{4}\times d^{3}\;{\left(M\cdot \mathrm{cm}\right)}^{-1}$$

where *d* is the edge length of the NCs in nanometers. The calculated value was (4.46 ± 0.22) × 10^7^ mol^−1^cm^−1^, which is fairly close to the values obtained from the aforementioned determination of extinction coefficients. Some difference may be caused by the overestimation of the NCs mass because some ligands still may remain at the NC surface. Using the determined mass absorption coefficient and NC molar weight, linear absorption cross-sections may be easily calculated. The linear absorption cross-sections were 4.94 × 10^−14^ cm^2^ and 4.86 × 10^−14^ cm^2^ for CsPbBr_3_ and CsPb_0.78_Cd_0.22_Br_3_ NCs, respectively. Recently, absorption cross-sections at 400 nm in the range of 1.3–3.4 × 10^−14^ cm^2^ were reported for CsPbBr_3_ NCs with sizes of 6.2–9.3 nm, [76,77,78,79]; our values are similar and expectedly slightly higher due to a larger NC size. This supports the applicability of the estimated concentration for the correct measurements of multiphoton absorption cross-sections.

Concentrations of NCs and Rhodamine 6G were determined from Beer’s law. For Rhodamine 6G, the known extinction coefficient (~40,000 mol^−1^cm^−1^) at 500 nm was used. [80,81].

## Appendix B. Multiphoton Absorption Cross-Section Calculation

Rhodamine 6G in ethanol was used as a standard for the 2PA cross-section calculation. The Rhodamine 6G cross-section was taken to be 128 ± 84 GM (10^−50^ cm^−4^·s·photon^−1^), as averaged from the literature data (taken at 800 nm with 100 fs pulse duration) [42,43,44]. To estimate the 2PA cross-section, one-photon excited PLQY was measured first. PLQY was measured against a standard, Rhodamine 6G in ethanol (PLQY ~0.9). PL spectra were measured using a Cary Eclipse spectrofluorometer (Varian Inc., Palo Alto, CA, USA). Absorption spectra were taken using a Shimadzu UV3600 spectrophotometer (Shimadzu corporation, Kyoto, Japan). PLQY was calculated using Equation (A4):

(A4) $$\varphi_{s}=\varphi_{r}\frac{I_{s}}{I_{r}}\frac{D_{r}}{D_{s}}{\left(\frac{n_{r}}{n_{s}}\right)}^{2},$$

where *φ*—one-photon excited PL quantum yield; *I*—PL spectra area, *D*—the optical density at the excitation wavelength, *n*—solution refractive index; indices *r* and *s* represent values for the reference and the sample, respectively.

Then, the 2PA cross-section was calculated using the Equation (A5):

(A5) $$\sigma_{s}^{2PA}\eta_{s}=\sigma_{r}^{2PA}\eta_{r}\frac{\varphi_{r}}{\varphi_{s}}\frac{I_{s}^{2P}}{I_{r}^{2P}}\frac{C_{r}}{C_{s}}\frac{n_{r}}{n_{s}},$$

where *σ^2PA^*—2PA cross-section (in GM); *φ*—one-photon excited PLQY; *η*—two-photon excited PLQY; *I*^2P^—two-photon excited PL spectra area, *C*—concentration (in M), *n*—solution refractive index; indices *r* and *s* represent values for the reference and the sample, respectively. Assuming that *φ* = *η*, the equation can be re-written as follows:

(A6) $$\sigma_{s}^{2PA}=\sigma_{r}^{2PA}{\left(\frac{\varphi_{r}}{\varphi_{s}}\right)}^{2}\frac{I_{s}^{2P}}{I_{r}^{2P}}\frac{C_{r}}{C_{s}}\frac{n_{r}}{n_{s}},$$

The 3PA absorption cross-section was calculated analogous to the 2PA cross-section with Rhodamine 6G in ethanol as a reference using the following equation:

(A7) $$\sigma_{s}^{3PA}=\sigma_{r}^{3PA}{\left(\frac{\varphi_{r}}{\varphi_{s}}\right)}^{2}\frac{I_{s}^{3P}}{I_{r}^{3P}}\frac{C_{r}}{C_{s}}\frac{n_{r}}{n_{s}},$$

where *σ^3PA^*—3PA cross-section (in GM); *φ*—one-photon excited PLQY; *I*^3P^—three-photon excited PL spectra area, *C*—concentration (in mol), *n*—solution refractive index; indices *r* and *s* represent values for the reference and the sample, respectively. The 3PA cross-section of Rhodamine 6G was taken as 3 × 10^−80^ cm^6^ s^2^ [45].
